# An experimental and theoretical approach to electrochemical sensing of environmentally hazardous dihydroxy benzene isomers at polysorbate modified carbon paste electrode

**DOI:** 10.1038/s41598-022-06207-6

**Published:** 2022-02-09

**Authors:** Pattan-Siddappa Ganesh, Sang-Youn Kim, Savas Kaya, Rajae Salim

**Affiliations:** 1 Interaction Laboratory, Advanced Technology Research Center, Future Convergence Engineering, Korea University of Technology and Education, Cheonan-si, 31253 Chungcheongnam-do Republic of Korea; 2 Department of Pharmacy, Health Services Vocational School, Sivas Cumhuriyet University, Sivas, 58140 Turkey; 3 Laboratory of Engineering, Organometallic, Molecular and Environment (LIMOME), Faculty of Science, University Sidi Mohamed Ben Abdellah, Fez, Morocco

**Keywords:** Theoretical chemistry, Environmental sciences, Natural hazards, Chemistry

## Abstract

It is well known that, surfactants provide a neutral, positive and/or negative charge on the electrode surface by forming a monolayer, which in turn affects the charge transfer and redox potential during the electroanalysis process. However, the molecular level understanding of these surfactant-modified electrodes is worth investigating because the interaction of the analyte with the electrode surface is still unclear. In this report, we used quantum chemical models based on computational density functional theory (DFT) to investigate the polysorbate 80 structure as well as the locations of energy levels and electron transfer sites. Later, the bare carbon paste electrode (bare/CPE) was modified with polysorbate 80 and used to resolve the overlapped oxidation signals of dihydroxy benzene isomers. The m/n values obtained at polysorbate**/**CPE was approximately equal to 1, signifying the transfer of same number of protons and electrons. Moreover, the analytical applicability of the modified electrode for the determination of catechol (CC) and hydroquinone (HQ) in tap water samples gave an acceptable recovery result. Overall, the application of DFT to understand the molecular level interaction of modifiers for sensing applications laid a new foundation for fabricating electrochemical sensors.

## Introduction

Among all the electroanalytical methods, voltammetry is one of the widely adopted instrumental techniques used for the electroanalysis of various electroactive molecules^1^. The credentials of these voltammetric techniques owing to its portability, simplicity, inexpensive instrument cost, rapid response, high sensitivity and selectivity^2,3^. Over the past few decades, the electroanalytical researchers used unmodified and/or chemically modified carbonaceous working electrodes, such as; carbon paste^4,5^, carbon nanotube paste^6,7^, graphite pencil^8,9^, glassy carbon^10^, and screen-printed carbon electrode^11^ for the determination of variety of electroactive molecules. The fabrication of new modified working electrodes is one of the attractive and curious topics of electrochemistry research. Among these carbonaceous working electrodes, carbon paste electrodes are progressively used in the electroanalysis of pharmaceuticals, neurochemicals, toxic molecules and metal ions^4,5,12^, due to quick and high sensitivity in the results.

Hydroquinone (HQ, 1,4-dihydroxybenzene) and catechol (CC, 1,2-dihydroxybenzene) are the toxic phenolic compounds, used as a basic feed stocks in manufacturing industries such as; paint, leather, pharmaceutical, pesticide, cosmetics and plastic industries^13,14^. These positional isomers of phenol are toxic to human beings and environment. Usually, these molecules coexist and it was difficult to detect them simultaneously by developing sensitive and selective methods^15^. So far, the quantitative methods like spectrophotometry^16,17^, synchronous fluorescence^18^, high performance liquid chromatography^19^, electrochemiluminescence^20^ and electroanalytical^13–15^ methods were established. Among these methods, electroanalytical methods are most preferable due to high sensitivity, quick response and selectivity^21,22^. Since, the redox peaks of these phenolic isomers, HQ and CC are broad and overlapped with each other at bare electrodes^14,15^, so many chemically modified electrodes such as poly(rutin)^22^, poly(pyrogallol red)^23^, covalent organic framework^13^, MOF-rGO^14^, gold nanoparticles mesoporous silica^15^, SBA-15 mesoporous silica^24^ and electrospun carbon nanofiber^25^ have been employed for their simultaneous voltammetric detection.

Among all the modification protocols and the chemically modified electrodes, recently, surfactant modified electrodes are widely used in the electrochemical sensor field^6,26^. This is due to such surfactant modified electrodes can enhance the electrocatalytic property, stability, eliminate the surface fouling, fast reaction rate and reproducibility in the results^27,28^. We can find many literature reports on the surfactant modified electrode and some of them are quoted, such as; CTAB-Cu-GR/CPE^29^, poly (CTAB)/MWCNTs/PGE^30^, CTABMCPE^26^, ISSM-CNT-PE^31^, CPE/CTAB^32^, TX100/CPE^33^, CTAB/MWCNTsP^34^ and SDBS-EGPE^35^. It is proposed that, these surfactants give positive or negative charge on the surface of electrode by forming a monolayer which in turn affects the charge transfer and redox potential during electroanalysis^33^. However, it’s worthwhile to study the molecular level understanding of these surfactant modified electrodes because the interaction of analyte and electrode surface was still unclear. The use of computational density functional theory (DFT)-based quantum chemical modelling to study the electron transfer sites of modifiers has received very little attention. However, a few excellent studies on the mediating mechanism of modifiers have recently been published^4,36–38^. Surfactants, mainly polysorbate 80 was a biocompatible excipient used in pharmaceutical formulations^39^. Polysorbate 80 is a synthetic non-ionic surfactant made up of fatty acid esters of polyoxyethylene sorbitan, while oleic acid is the predominant fatty acid, other fatty acids such as linoleic or palmitic acid may be present^40^. As a result, polysorbate 80 is typically a complex chemical mixture of different fatty acid esters, with oleic acid accounting for more than 58% of the overall composition^39,41^. On the other hand, polysorbate 80 is primarily composed of polyoxyethylene-20-sorbitan monooleate, which is structurally identical to polyethylene glycols^42^. In this work, polysorbate 80 was used as a modifier to improve the number of active sites for electron transfer at the carbon paste electrode interface. The increased voltammetric signals at surfactant modified electrodes are well understood to be due to a greater number of analyte interactions. As a result, such electrodes can logically be considered better electrochemical sensors than bare carbon paste electrode (bare CPE). To fully comprehend this electrochemical phenomenon, a deeper understanding is needed. A study from the perspective of conceptual-DFT based on quantum chemical modelling is one approach to this end. The DFT studies of the polysorbate-80 modified carbon paste electrode (polysorbate/CPE) interface are significant because they can reveal the polysorbate 80 structure as well as the locations of energy levels and electron transfer sites. To the best of our knowledge, there is no literature report on this topic. As expected, the voltammetric signals obtained at fabricated polysorbate/CPE is more superior as compared to bare/CPE. Moreover, the overlapped oxidation signal of CC and HQ was resolved at polysorbate/CPE, which reflects the effective and interference free electrochemical sensing of the dihydroxy benzene isomers. Overall, here we showed that, understanding the electron transfer sites of modifiers through quantum chemical modelling, set a new basis for the fabrication of electrochemical sensing platforms and predicting the sensing mechanisms.

## Experimental part

### Reagents and instrumentation

Silicone oil (CAS: 63148-62-9) and graphite powder (≥ 99.99%, CAS:7782-42-5) (average particle size < 45 μM) were procured from Sigma-Aldrich and used for constructing the carbon paste electrode (CPE). NaH_2_PO_4_•2H_2_O (CAS: 13472-35-0) and Na_2_HPO_4_ (CAS: 7558-79-4) was used to prepare the phosphate buffer solution (PBS) of desired concentration (0.2 M) and required pH. Hydroquinone (HQ) (≥ 99%, CAS: 123-31-9), catechol (CC) (≥ 99%, CAS: 120-80-9), potassium ferrocyanide (≥ 99.95%, CAS: 14459-95-1), potassium ferricyanide (≥ 99%, CAS: 13746-66-2), potassium chloride (≥ 99%, CAS:7447-40-7) and polysorbate 80 (CAS: 9005-65-6) were received from Sigma-Aldrich and the solution of respective concentration was prepared in double distilled water. All the chemicals used were of analytical grade and the respective stock solutions were prepared without any additional treatment. All electrochemical testing’s performed with an electrochemical workstation (CHI660D). The three-electrode compartment comprised of a reference, counter and working electrode were used, namely; saturated calomel electrode (SCE), platinum wire and bare/CPE or polysorbate/CPE respectively. All the potential values obtained were reported versus SCE at an ambient temperature.

### Fabrication of the working electrodes

The bare carbon paste electrode (bare/CPE) was prepared by homogeneously mixing the graphite powder and a binder (silicone oil) in a 70:30 ratio^23^. The obtained uniform paste was then filled into the end of the Teflon hole and then polished on a smooth paper. Copper wire was inserted into the end of the Teflon tube for electrical contact. A different amount of polysorbate-80 solution (25.0 mM) was drop casted on to the surface of bare carbon paste electrode and allowed to stand for five minutes (at room temperature) to optimise the polysorbate/CPE. Finally, the electrode was rinsed with distilled water to remove any excess polysorbate-80 solution^6,33^.

### Computational methods

The quantum chemical calculation is an important method which provide us a large information about structural properties, and therefore lead us to predict their reactivity^43^. DFT method based on B3LYP/6-31G (d, p) base was performed using Gaussian 09 program in order to estimate the reactivity of polysorbate 80 molecule. It is well known that a several descriptors of chemical reactivity such as chemical electronegativity (*χ*), hardness (*η*) and softness (*σ*) was can be extracted according to Parr and Pearson using the following equations^44^.

1 $$ {\text{Electronegativity }}\chi = \left[ {\frac{\partial E}{{\partial N}}} \right]_{\nu (r)} = - \left( {\frac{I + A}{2}} \right) $$

2 $$ {\text{Hardness }}\eta = \frac{1}{2}\left[ {\frac{{\partial^{2} E}}{{\partial N^{2} }}} \right]_{\nu (r)} = \frac{I - A}{2} $$

3 $$ {\text{Softness }}\sigma = 1/\eta $$

where the ionization energy $I = - E_{HOMO}$ and electron affinity $A = - E_{LUMO}$, according to Koopmans Theorem^45^. The solvent effect investigation was included using conductor-like polarizable continuum model (CPCM).

On the other hand, the electro-accepting power (*ω*^+^) and electro-donating power (*ω*^*−*^) parameters which is conditional on ionization energy and electron affinity concepts can predict the electron donating and electron accepting abilities of studied chemical species (Eqs. 4 & 5)^46^:

4 $$ {\text{electro}} - {\text{accepting power }}\omega^{ + } = (I + 3A)^{2} /(16(I - A)) $$

5 $$ {\text{electro}} - {\text{donating power }}\omega^{ - } = (3I + A)^{2} /(16(I - A)) $$

The Fukui function indicates the tendency of a molecule to give or obtain electrons. On the other hands, these functions have been modeled to detect the most nucleophilic interactions in a molecule^47^. The electrophilic ($f_{k}^{ - }$) and nucleophilic ($f_{k}^{ + }$) attacks are calculated using Eqs. (6 & 7):

6 $$ f_{k}^{ + } = P_{k} \left( {N + 1} \right) - P_{k} \left( N \right){\text{Nucleophilic attack}} $$

7 $$ f_{k}^{ - } = P_{k} \left( N \right) - P_{k} \left( {N - 1} \right){\text{Electrophilic attack}} $$

where $P_{k}$ is the natural population for atom k site in the cationic (*N − *1), anionic (*N* + 1) or neutral molecule (*N*).

## Result and discussions

### Calibration and characterisation of polysorbate/CPE

**Figure 1:**
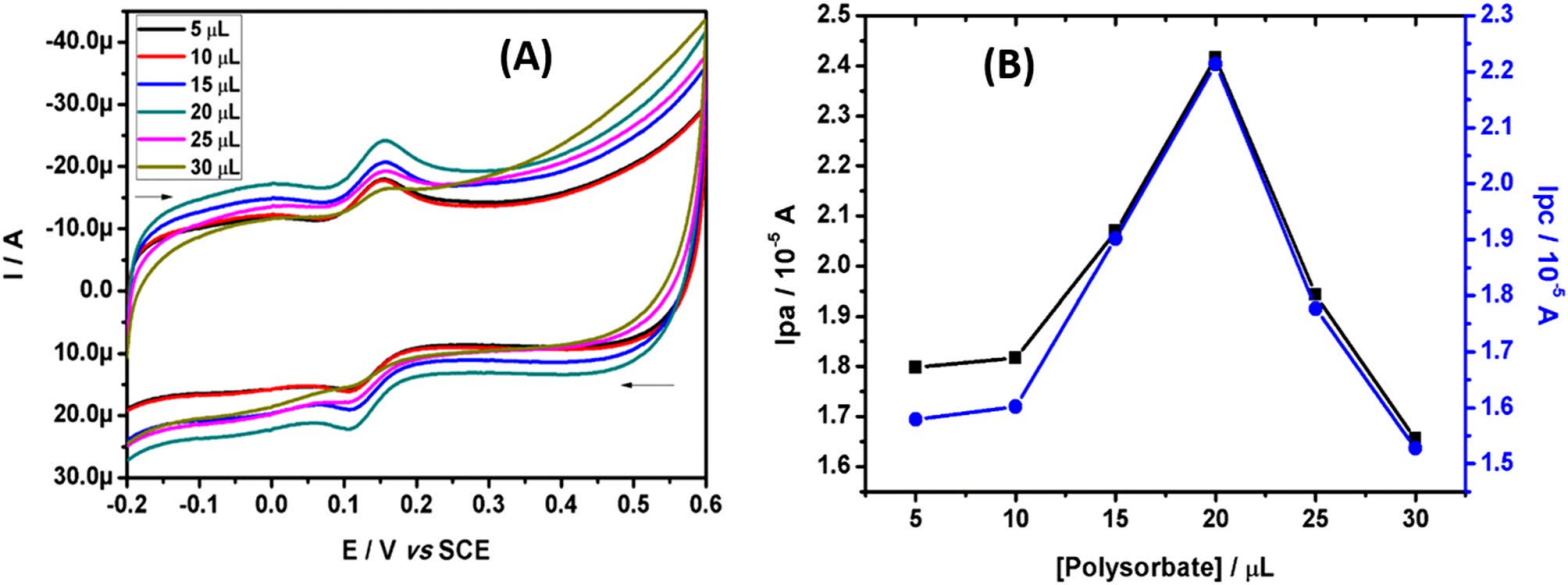
(**A**) CVs recorded for 5.0 μM CC at polysorbate/CPE with different immobilization volume of polysorbate 80 solution. (**B**) Graph of peak current of CC versus volume of polysorbate 80.

The different volume of polysorbate-80 solution (25.0 mM) was used as modifier and applied for the electroanalysis of CC. As the volume of modifier increases the peak current response of CC increased at first till 20.0 μL, later the analyte showed the downfall in the current response as showed in Fig. 1A. This may be due to the surface hindrance of polysorbate 80 long chain^33^. It was clear from the Fig. 1B that, the peak current of CC was maximum at 20.0 μL volume of polysorbate 80 solution and the ratio of anodic peak current (Ipa) to cathodic peak current (Ipc) was found to be 1.04 (Ipa/Ipc ≈ 1), which is a typical characteristic voltammogram for the reversible electrooxidation of CC. Therefore, 20.0 μL volume was used for the fabrication of polysorbate**/**CPE and employed for the analysis of hazardous dihydroxy benzene isomers. According to previous reports, the surface area of the working electrode was calculated by the Randles–Sevcik equation^23^. The calculated surface area of polysorbate**/**CPE and bare/CPE was 0.03913 and 0.02821 cm^2^ respectively.

**Figure 2:**
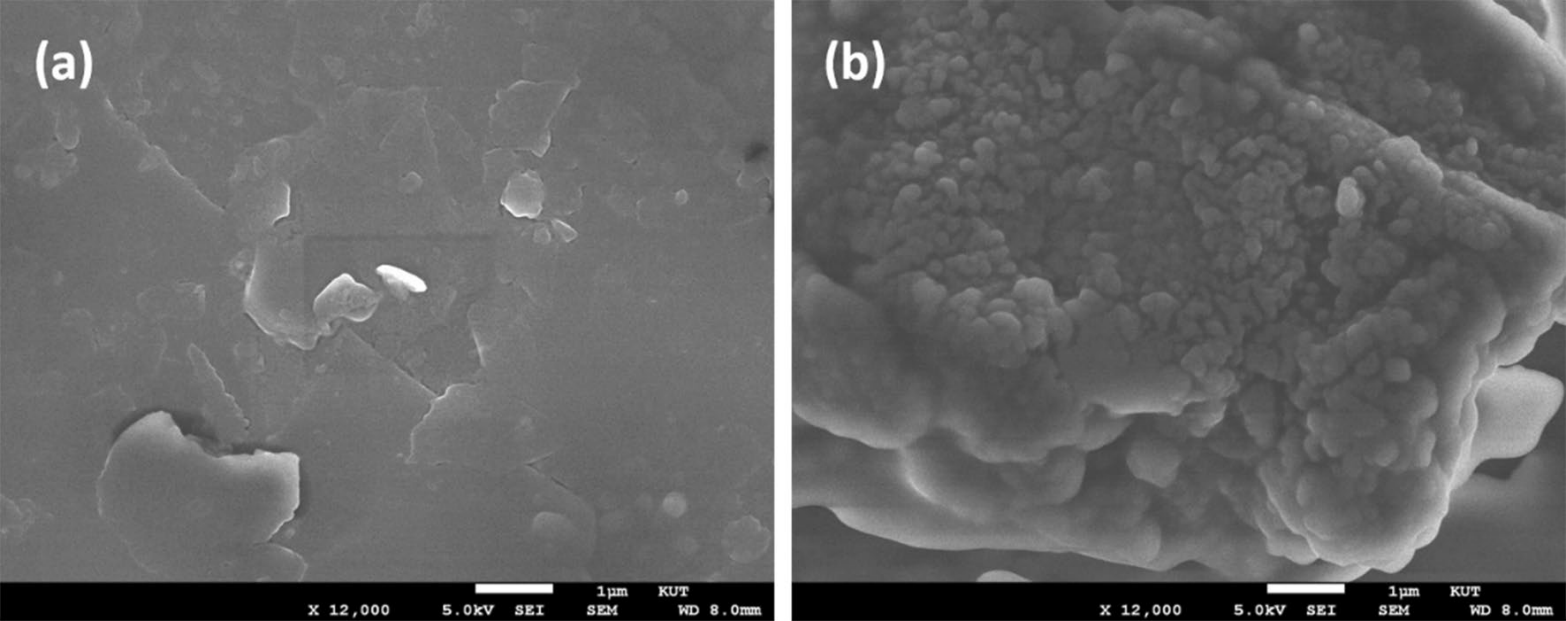
SEM images of bare/CPE (**a**) and polysorbate/CPE (**b**).

The surface texture of bare/CPE (a) and polysorbate**/**CPE (b) can be better understood by comparing the scanning electron microscopy (SEM) images with each other as depicted in Fig. 2. It can be observed that bare/CPE has predominated with graphite flakes with irregular arrangement, which is more favourable for the adsorption of surfactants. Whereas polysorbate**/**CPE shows the formation of active sites, which filled the gap between the graphite flakes due to the adsorption of polysorbate-80 on the surface of carbon paste electrode. Moreover, the polysorbate/CPE consists of many ridges and valleys as compared to bare/CPE. This reflects the successful modification of bare electrode by polysorbate-80 surfactant.

### DFT studies

**Figure 3:**
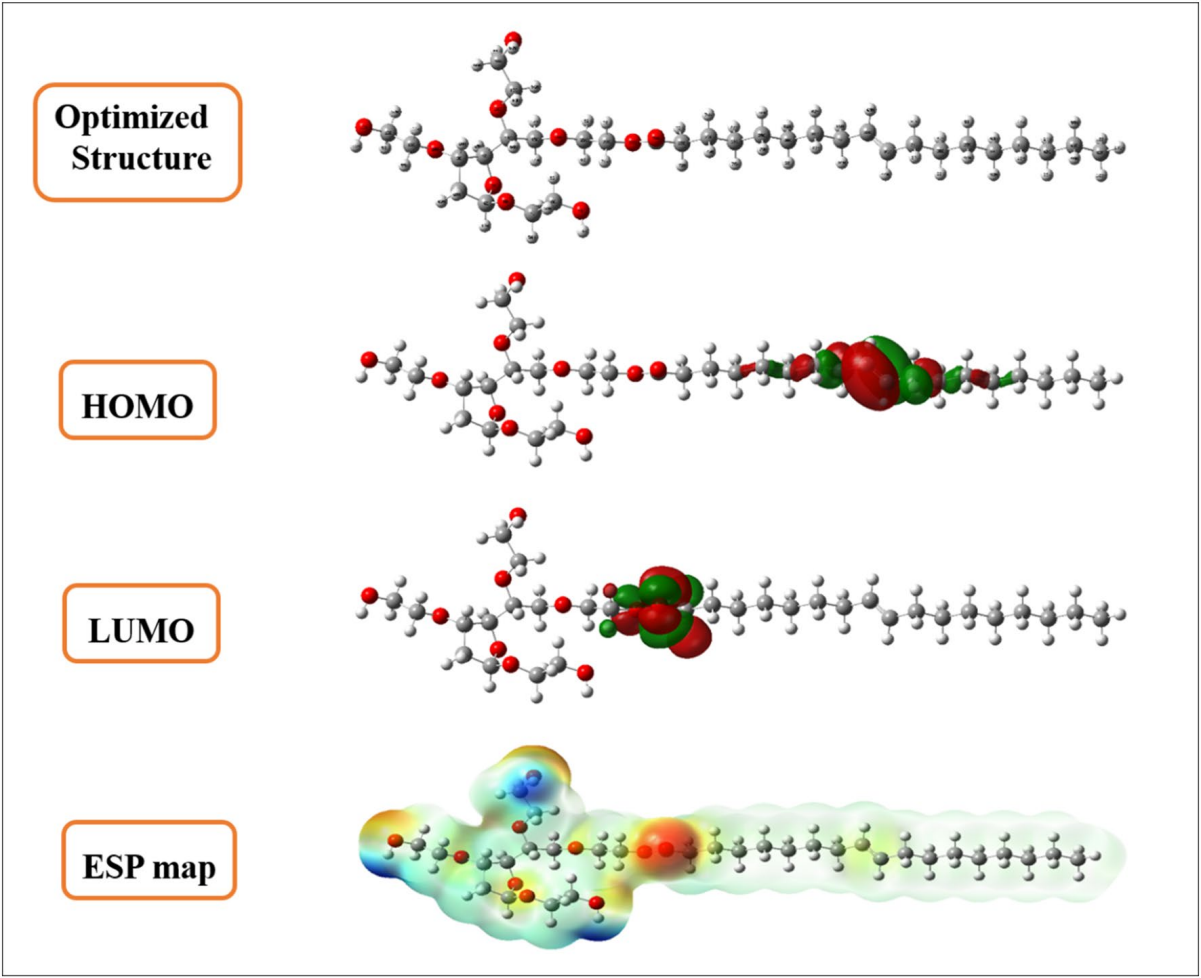
Optimized structures, HOMO & LUMO, and ESP maps for polysorbate 80 molecule in aqueous phase.

Theoretical methods are considered as new efficient and inexpensive method to describe the molecules reactivity from the calculations of some chemical descriptors^48^. This approach was done using density functional theory at B3LYP with 6-31G (d, p) basis set. The optimized geometries of polysorbate 80 molecule as well as their frontier molecular orbitals (HOMO and LUMO), and ESP map are presented in Fig. 3. While, the quantum chemical descriptors extracted and regrouped in Table 1.

**Table 1:** Quantum chemical descriptors for polysorbate 80 in aqueous phases.

Descriptors	E_HOMO_ (eV)	E_LUMO_ (eV)	σ (eV^−1^)	Ƞ (eV)	χ (eV)	ω +	ω−
Polysorbate 80	− 6.3553	5.4777	0.1690	5.9165	0.4387	0.5364	0.9752

It can be seen from Fig. 3 that the HOMO distribution density localized on the C=C group in the carbonic chain while the LUMO density localized particularly in the –O–O– group in the carbonic chain. Also, Electrostatic potential surface (ESP) is another way that can give us information about the electrophilic active sites existed in the chemical species. The ESP maps result leads us to suggest that the electrophilic active site is localized specially on –O–O– group since showing a red-yellow color. On the other sense, these sites are able to change and transfer electron with the surface of studied working electrode^49^.

It’s well known that the high values of HOMO orbital energy define the ability of molecule to donate electron. While, the low LUMO energy values explain the acceptor ability^50^. In our case, polysorbate 80 compound goes with the same trend providing a high reactivity to remove an electron from the last occupied orbital to low unoccupied one. According to Kaya et al.^51^, the chemical hardness can be defined as the resistance towards electron cloud polarization or deformation of chemical species. In other sense, the molecules considered more reactive when it having a small hardness value and a high softness value. The hardness and softness obtained values goes with the same tendency. Gazquez parameters calculated for polysorbate 80 showed an electro-donating ability with a donor capacity values ω^−^ = 0.9752 eV (oxidation process) and acceptor capacities of ω^+^  = 0.5364 eV (reduction process)^36^. Finally, it can be concluded that quantum global descriptors that polysorbate 80 molecular structure shows a high reactivity performance confirming the high interaction with the working electrode, and explaining the adsorption of this molecule onto the working electrode surface.

It can be seen from Table 2 Fukui indices that the calculated values of $f_{k}^{ + }$ for polysorbate 80 are typically localized on C24, C34, C33, and O26 which leads us to suggest that these atoms are able to form a back bond by accepting the electron comes from the working electrode surface. On the other hand, C33, C34, O16 and O10 are the most active sites for the electrophilic attacks since recording the highest values of $f_{k}^{ - }$ implying that suitable to donor–acceptor interactions and thus facilitate the adsorption of polysorbate 80 on the materials surface^52^. These results were confirming the results obtained by frontier orbital molecular (HOMO, LUMO) and ESP map. Finally, the studied molecule can be used to modify the working electrode surface for electrochemical sensors applications.

**Table 2:** Most active sites of $f_{k}^{ + }$,$f_{k}^{ - }$ for polysorbate 80 in aqueous phases.

Atoms	*P (N)*	*P (N−*1*)*	*P (N* + 1*)*	$f_{k}^{ + }$	$f_{k}^{ - }$
O 6	8.5859	8.5382	8.5938	0.0078	0.0476
C 8	6.1078	6.1118	6.1184	0.0106	− 0.0040
O 10	8.5844	8.5122	8.5919	0.0075	**0.0722**
O 13	8.7902	8.7709	8.7992	0.0090	0.0193
O 16	8.5909	8.5106	8.5926	0.0017	**0.0802**
O 19	8.7889	8.7642	8.7892	0.0003	0.0247
O 23	8.5680	8.5624	8.5894	0.0213	0.0056
C 24	5.1656	5.1624	5.2730	**0.1074**	0.0032
O 26	8.6021	8.5808	8.6639	**0.0618**	0.0212
C 32	6.4893	6.5115	6.4756	− 0.0136	− 0.0222
C 33	6.2213	6.0926	6.3100	**0.0887**	**0.1286**
C 34	6.2205	6.0881	6.3189	**0.0984**	**0.1323**
C 35	6.4899	6.5122	6.4753	− 0.0145	− 0.0223

### The electrochemical behaviour of CC and HQ at bare/CPE and polysorbate/CPE

**Figure 4:**
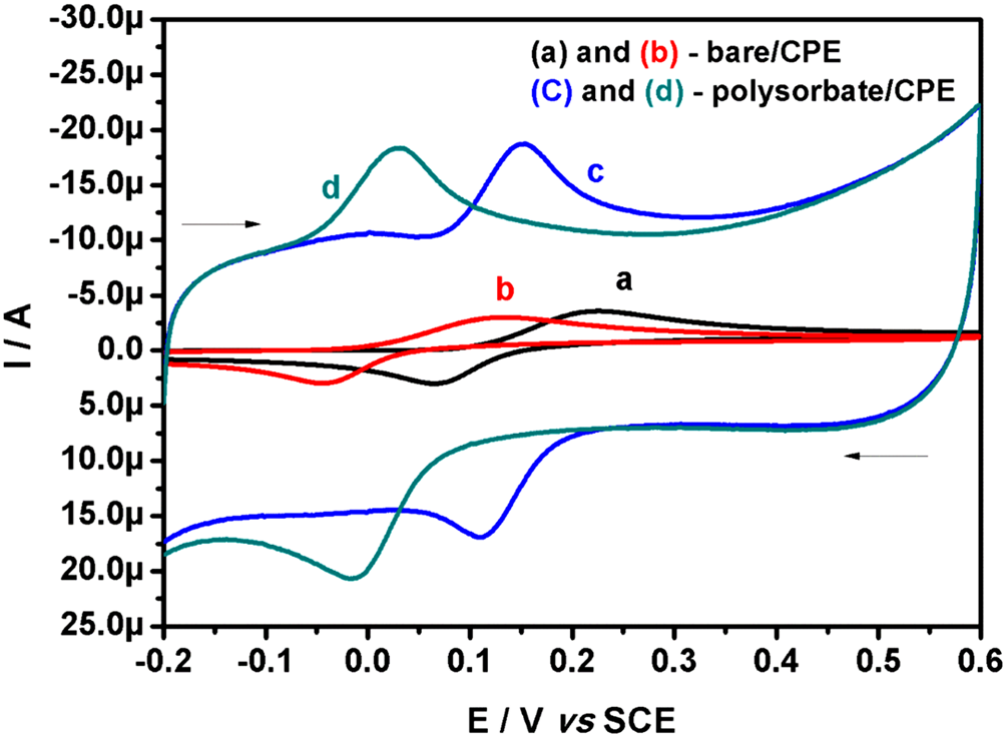
CVs of 20.0 μM CC (curve **a**) and 20.0 μM HQ (curve **b**) at bare/CPE, the improved CVs observed at polysorbate/CPE for CC (curve **c**) and HQ (curve **d**) in buffer (0.2 M PBS; pH 7.4) with 0.05 Vs^−1^ scan rate.

**Figure 5:**
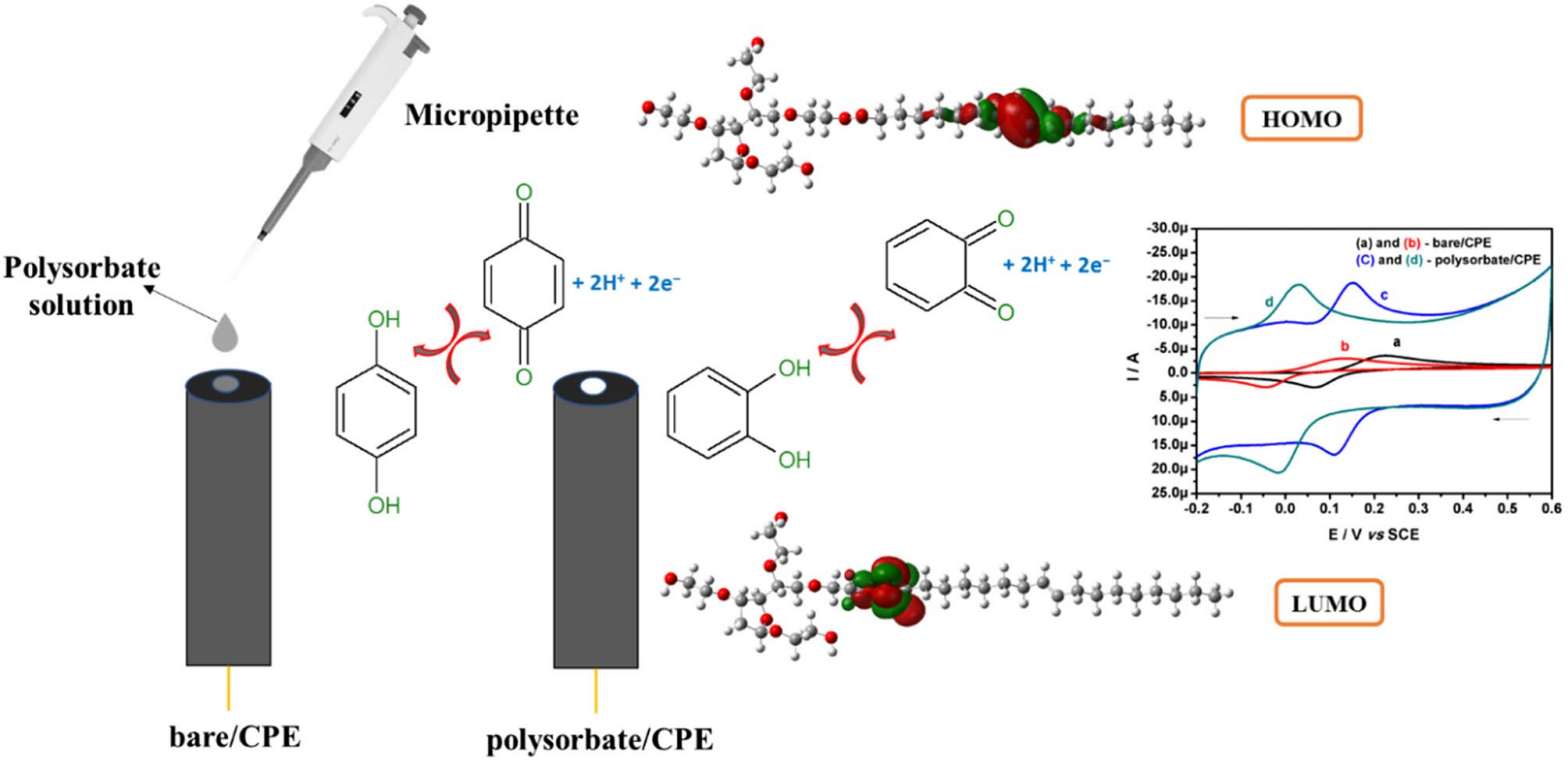
Fabrication of polysorbate/CPE and its electrocatalytic interaction with CC and HQ.

The electrochemical behaviour of CC and HQ in PBS (0.2 M, pH 7.4) was studied at bare/CPE and polysorbate/CPE by CV technique as shown in Fig. 4. The CC (20.0 μM, curve a) and HQ (20.0 μM, curve b) showed a broad voltammogram with poor response at bare/CPE and the oxidation potential was located at 0.228 V and 0.126 V respectively. The peak potential difference (ΔEp) of CC was 0.158 V and that of HQ was 0.166 V, this result clearly exhibits the poor performance of the bare/CPE. However, at polysorbate/CPE the minimisation of overpotential for CC (curve c) and HQ (curve d) oxidation was observed and the oxidation potentials were observed at 0.151 V and 0.027 V for CC and HQ respectively. Moreover, the ΔEp value were found to be 0.038 V for CC and 0.040 V for HQ. There is almost a tenfold enhancement in current signal at polysorbate/CPE as compared with bare/CPE. We calculated the ratio of Ipa to Ipc for both CC and HQ at polysorbate/CPE, and were found to be approximately equal to 1, which is a characteristic voltammogram of a typical reversible system. Therefore, these results confirm the superiority of the polysorbate/CPE towards the electrochemical detection of hazardous CC and HQ. For better understanding of the fabrication of polysorbate/CPE and its electrocatalytic interaction with CC and HQ the readers may refer the Fig. 5.

### Effect of scan rate

**Figure 6:**
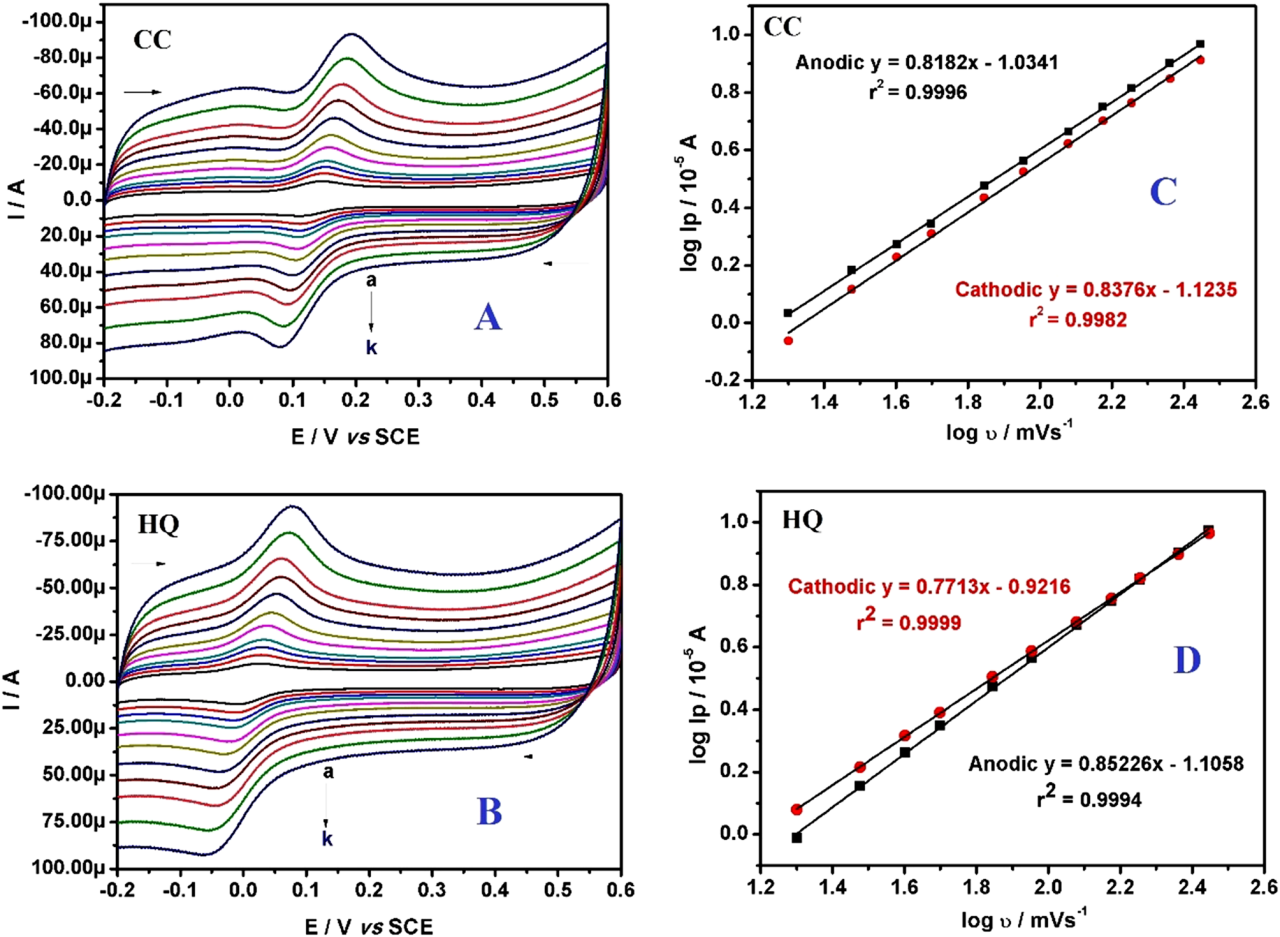
(**A**) CVs on impact of scan rates (a–k: 20, 30, 40, 50, 70, 90, 120, 150, 180, 230, 280 mVs^−1^) on electrooxidation of 20.0 μM CC at polysorbate/CPE in buffer (0.2 M PBS; pH 7.4). (**B**) CVs on impact of scan rates (a–k: 20, 30, 40, 50, 70, 90, 120, 150, 180, 230, 280 mVs^−1^) on electrooxidation of 20.0 μM HQ at polysorbate/CPE in buffer (0.2 M PBS; pH 7.4). (**C**) Graph of log Ip versus log υ of CC. (**D**) Graph of log Ip versus log υ of HQ.

In order to extract the information on the electrode phenomenon, cyclic voltammograms for 20.0 μM CC and 20.0 μM HQ in PBS at pH 7.4 was carried out for varying scan rate. From the Fig. 6A,B it was noticed that, as the scan rate increases, there was an enhancement in the corresponding redox current signal with a slight shift in peak potentials. This observation was in accordance with Randles–Sevcik’s relationship. On the other side, the graph of logarithm of peak current (log Ip) versus logarithm of scan rate (log υ) for both CC and HQ was constructed as shown in Fig. 6C,D respectively. The corresponding linear regression equations are noted as follows:

For CC:

log Ipa (10^–5^ A) = 0.8182 log υ (mVs^−1^)—1.0341 (r^2^ = 0.9996) (for anodic).

log Ipc (10^–5^ A) = 0.8376 log υ (mVs^−1^)—1.1235 (r^2^ = 0.9982) (for cathodic).

For HQ:

log Ipa (10^–5^ A) = 0.8522 log υ (mVs^−1^)—1.1058 (r^2^ = 0.9994) (for anodic).

log Ipc (10^–5^ A) = 0.7713 log υ (mVs^−1^)—0.9216 (r^2^ = 0.9999) (for cathodic).

**Figure 7:**
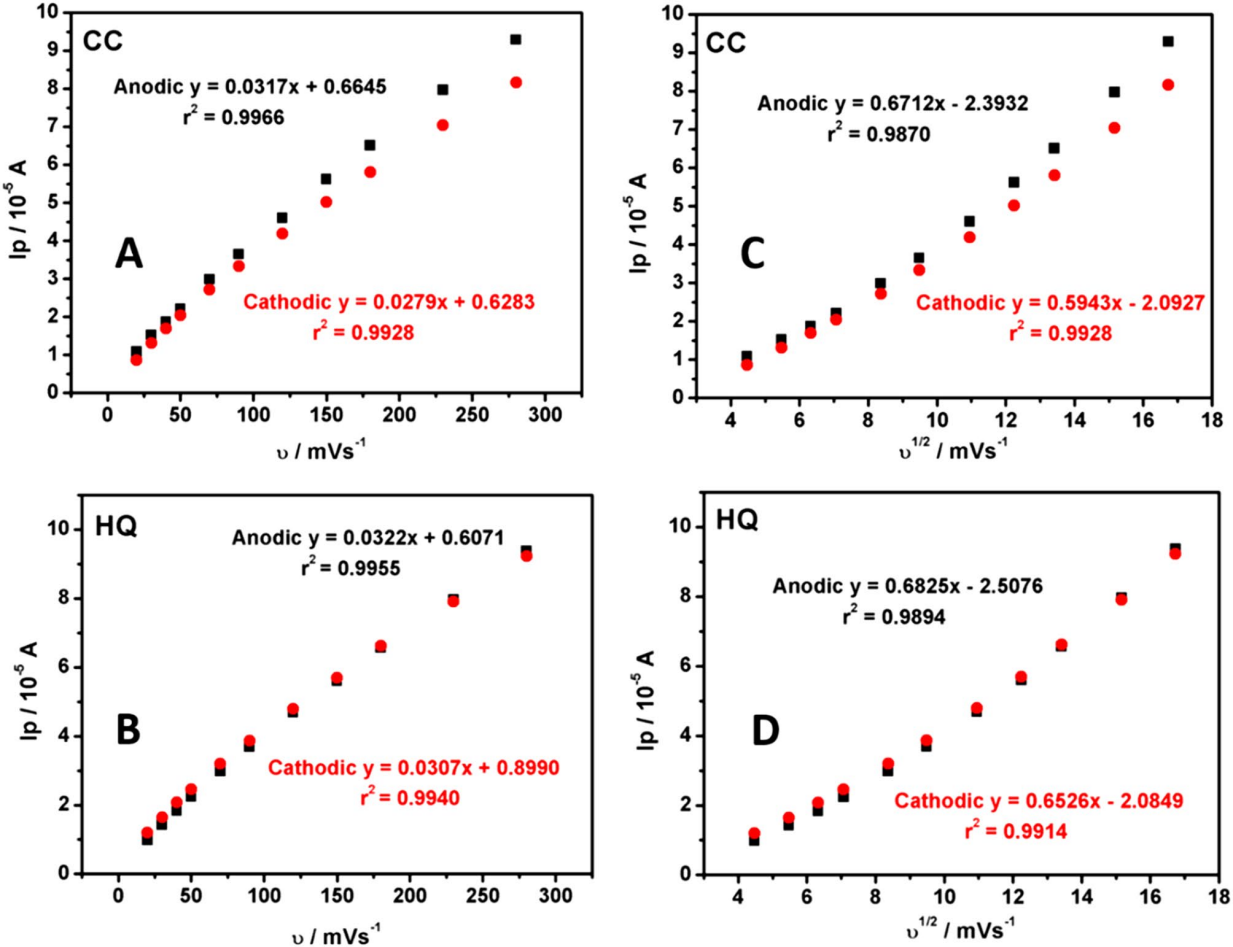
(**A**) Graph of Ip versus υ for CC. (**B**) Graph of Ip versus υ for HQ. **(C)** Graph of Ip versus υ^1/2^ for CC. (**D**) Graph of Ip versus υ^1/2^ for HQ.

The obtained slope values of 0.8182 and 0.8376 for anodic and cathodic response for CC, and 0.8522 and 0.7713 corresponding to anodic and cathodic response of HQ agree with the theoretical values of 1.0 (adsorption controlled)^23^. This result was again confirmed by the linear plots of peak current (Ip) versus scan rate (υ) and Ip versus square root of scan rate (υ^1/2^) for both CC and HQ (see Fig. 7). It can be seen that, a clear linearity was obtained for Ip versus υ of both CC and HQ, the linear regression equations are as below:

For CC:

Ipa (10^–5^ A) = 0.0317 υ (mVs^−1^) + 0.6645 (r^2^ = 0.9966) (for anodic).

Ipc (10^–5^ A) = 0.0279 υ (mVs^−1^) + 0.6283 (r^2^ = 0.9928) (for cathodic).

For HQ:

Ipa (10^–5^ A) = 0.0322 υ (mVs^−1^) + 0.6071 (r^2^ = 0.9955) (for anodic).

Ipc (10^–5^ A) = 0.0307 υ (mVs^−1^) + 0.8990 (r^2^ = 0.9940) (for cathodic).

In both CC and HQ scan rate study, the obtained correlation coefficient (r^2^) values suggest the typical adsorption dominated process^53^. This behaviour agrees with the previous report^24,54^. According to the reported formulae (8)^55,56^, the heterogeneous rate constant (k^0^) values were calculated for the electrooxidation of both CC and HQ. The obtained values were tabulated in Table 3.

8 $$ \Delta {\text{Ep}} = 201.39 \log ({\text{u}}/{\text{k}}^{0}) - 301.78$$

**Table 3:** k^0^ values calculated for the electrooxidation of CC and HQ at polysorbate 80/CPE.

υ mV/s	ΔEp/V		k^0^/s	
	CC	HQ	CC	HQ
20	0.0373	0.0364	0.4144	0.4006
30	0.0384	0.0413	0.6139	0.6042
40	0.0433	0.0492	0.8142	0.7995
50	0.0472	0.0551	0.9886	0.9992
70	0.0512	0.0629	1.1501	1.1621
90	0.0570	0.0757	1.3700	1.3825
120	0.0630	0.0896	1.5102	1.5421
150	0.0689	0.1043	1.6986	1.7321
180	0.0768	0.1044	1.9210	2.1024
230	0.0985	0.1230	2.1862	2.3420
280	0.1141	0.1387	2.4211	2.6421

### Influence of pH

**Figure 8:**
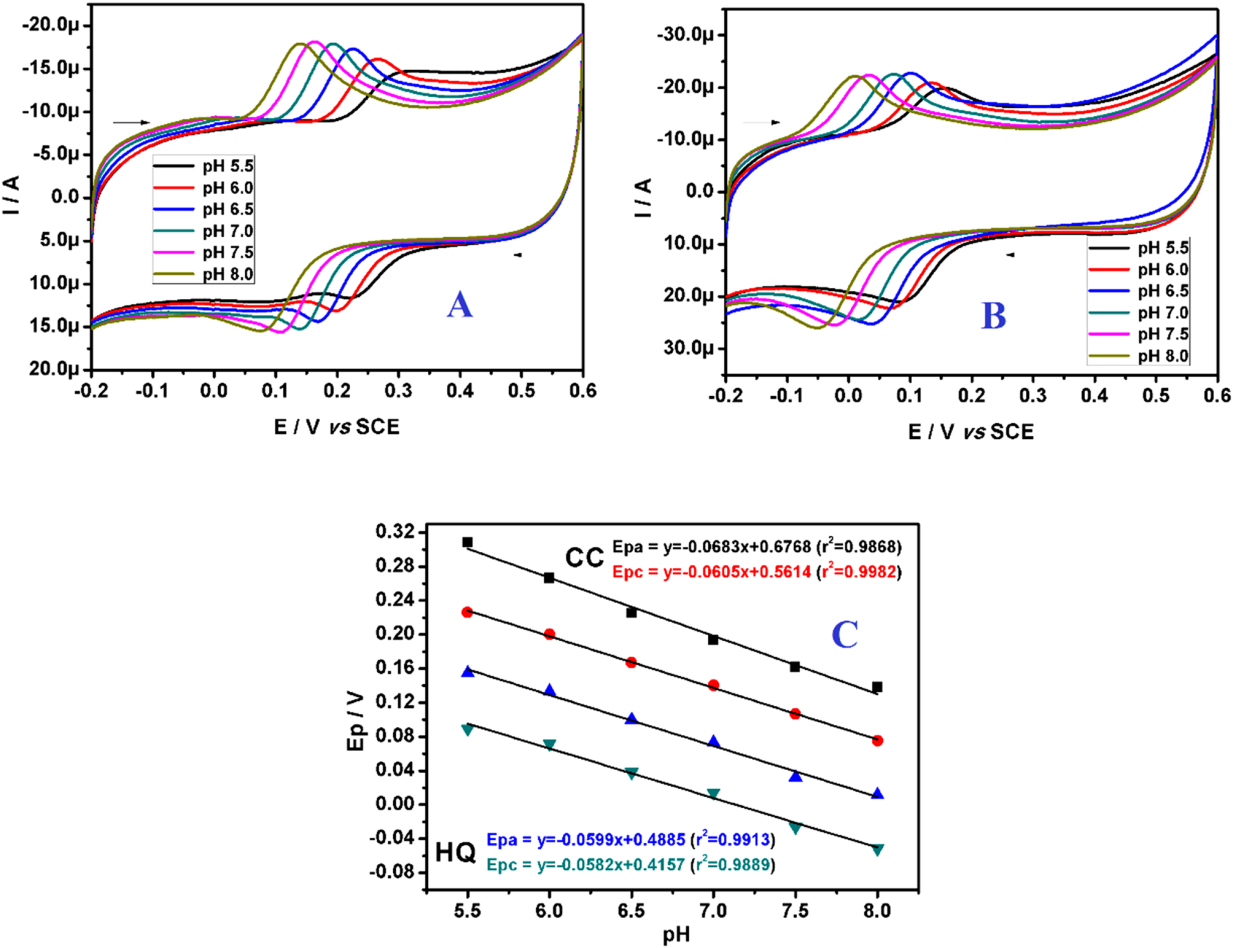
(**A**) CVs obtained for electrooxidation of 20.0 μM CC at polysorbate/CPE in buffer of different pH (5.5 to 8.0) at 0.05 Vs^−1^scan rate. (**B**) CVs obtained for electrooxidation of 20.0 μM HQ at polysorbate/CPE in buffer of different pH (5.5 to 8.0) at 0.05 Vs^−1^scan rate. (**C**) Graph of peak potentials of CC and HQ versus pH of buffer (PBS).

The electrochemical investigations carried out in aqueous medium are depends on the solution pH^57,58^. The Fig. 8A,B shows the influence of solution pH (PBS) on the electrooxidation of 20.0 μM CC and 20.0 μM HQ respectively, which was studied by CV technique. It is very clear that as the solution pH increases the oxidation–reduction potentials shift towards the least potential scale. The graph of peak potential and solution pH was showed in Fig. 8C; the linear regression equation can be expressed as below:

For CC:

Epa (V) = − 0.0683pH + 0.6768 (r^2^ = 0.9868) (versus SCE) (for anodic).

Epc (V) =  − 0.0605pH + 0.5614 (r^2^ = 0.9982) (versus SCE) (for cathodic).

For HQ:

Epa (V) =  − 0.0599pH + 0.4885 (r^2^ = 0.9913) (versus SCE) (for anodic).

Epc (V) =  − 0.0582pH + 0.4157 (r^2^ = 0.9889) (versus SCE) (for cathodic).

By using Nernst formulae, we calculated the m/n values^59,60^ given in Eq. (9). Where, m and n are the number of protons and electrons respectively. The R, T and F have their usual significance.

9 $$\frac{\mathrm{dEp}}{\mathrm{dpH}}=\frac{2.303\mathrm{mRT}}{\mathrm{nF}}$$

**Figure 9:**
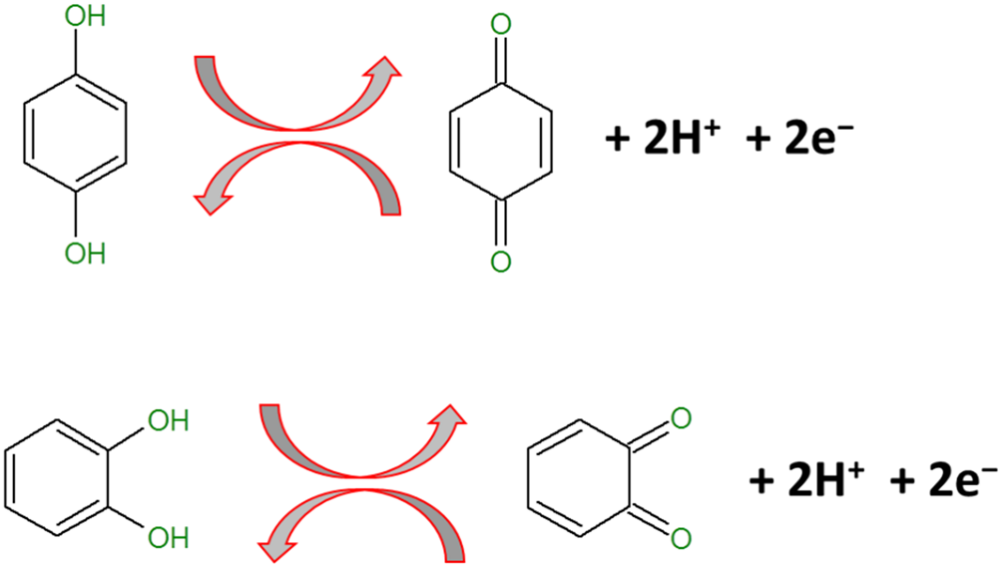
Electrooxidation mechanism of HQ and CC.

The m/n values were calculated to be 1.155 and 1.023 for anodic and cathodic response of CC respectively, on the other side, the m/n values obtained for anodic and cathodic response of HQ was 1.012 and 1.011 respectively. This result confirms that, at polysorbate**/**CPE electrooxidation of CC and HQ involves transfer of identical number of protons and electrons^61^. It was proposed that, the two oxygen-hydrogen bonds of phenolic hydroxyl moieties lead to destruction, in the interim CC and HQ lose two protons and two electrons forming a quinonoid structure and converse for reduction as showed in Fig. 9^62^.

### Effect of varying concentration

**Figure 10:**
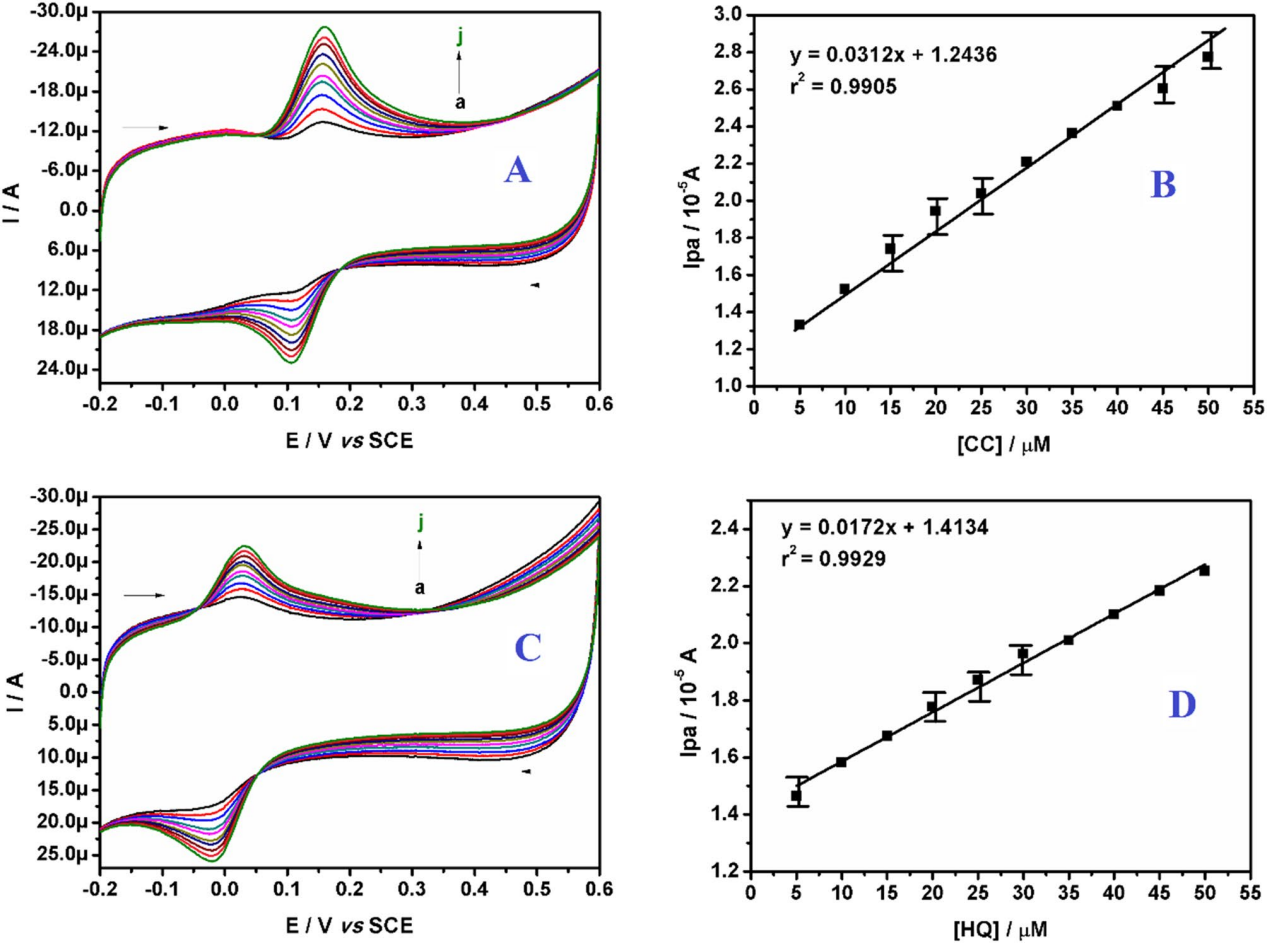
(**A**) CVs of polysorbate/CPE with different concentration of CC (5.0 to 50.0 μM) in buffer (0.2 M PBS; pH 7.4). (**B**) Linear plot of Ipa versus concentration of CC. **(C)** CVs of polysorbate/CPE with different concentration of HQ (5.0 to 50.0 μM) in buffer (0.2 M PBS; pH 7.4). (**D**) Linear plot of Ipa versus concentration of HQ.

The concentration studies of both the targeted analytes were carried out at polysorbate**/**CPE by cyclic voltammetry technique. Figure 10A,C showed an increase in current signal due to the increase in concentration of CC and HQ respectively. The linearity graphs of Ipa versus concentration of CC and HQ were established in the Fig. 10B,D respectively. The corresponding linear regression equations are as follows:

Ipa (10^-5^A) = 0.0312 (C_0_ μM/L) + 1.2436, (r^2^ = 0.9905) (for CC).

Ipa (10^-5^A) = 0.0172 (C_0_ μM/L) + 1.4134, (r^2^ = 0.9929) (for HQ).

The limit of detection (LOD) and limit of quantification (LOQ) was calculated by using the formulae (10) and (11), where S is the standard deviation of the six blank measurements and M is the slope of the calibration graph^63^.

10 $$ {\text{LOD}} = {\text{3S}}/{\text{M}} $$

11 $$ {\text{LOQ}} = {1}0{\text{S}}/{\text{M}} $$

The LOD and LOQ value of CC was calculated to be 0.91 μM and 3.03 μM respectively, while the LOD and LOQ for HQ was 0.82 μM and 2.73 μM and which are relatively lower than the previous reports as tabulated in Table 4^64–74^.

**Table 4:** Comparison of LOD obtained for CC and HQ at polysorbate/CPE with other modified electrodes, method and pH of the supporting electrolyte used.

Sl No	Working electrode	LOD (μM)		Method	pH	Ref
		**CC**	**HQ**			
1	Silsesquioxane/MCPE	10.0	10.0	DPV	7.0	^64^
2	*p*-Phe modified electrode	0.7	1.0	DPV	5.0	^65^
3	LDHf/GCE	1.2	9.0	DPV	6.5	^66^
4	PASA/MWNTs/GCE	1.0	1.0	DPV	6.0	^67^
5	PEDOT/GO	1.6	1.6	DPV	6.0	^68^
6	Cu(Sal-β-Ala) (3,5-DMPz)2]/SWCNTs/GCE	3.5	1.46	DPV	6.0	^69^
7	CNx/GCE	2.71	1.20	LSV	4.7	^70^
8	Co_3_O_4_/MWCNTs/GCE	8.5	5.6	DPV	8.0	^71^
9	MWCNT–NF–PMG	31.0	18.1	CV	1.5	^72^
10	Poly (benzoguanamine)/MCPE	2.55	3.84	CV	7.4	^73^
11	NiO/CNT/GCE	2.5	2.5	DPV	7.0	^74^
12	Polysorbate/CPE	0.91	0.82	CV	7.4	This work

### Simultaneous determination of CC and HQ at polysorbate/CPE

**Figure 11:**
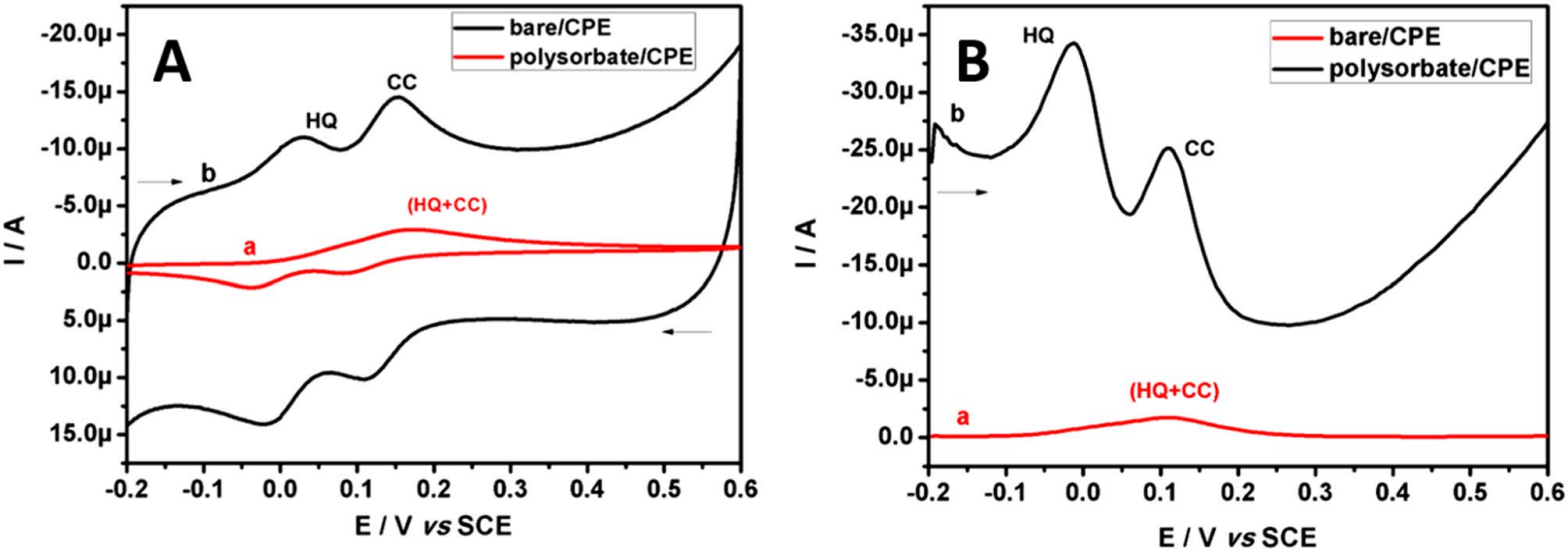
(**A**) CVs recorded for the simultaneous determination of equimolar (10.0 μM) binary mixture of CC and HQ at bare/CPE and polysorbate/CPE in buffer (0.2 M PBS; pH 7.4) with 0.05 Vs^−1^ scan rate. (**B**) DPVs recorded for the simultaneous determination of equimolar (50.0 μM) binary mixture of CC and HQ at bare/CPE and polysorbate/CPE in buffer (0.2 M PBS; pH 7.4).

Since the isomers of dihydroxybenzene have the same chemical structure, the bare/CPE fails to resolve the oxidation potentials of these isomers. On the other hand, the main assignment of the polysorbate/CPE is to discriminate the merged oxidation potentials of CC and HQ, which is practically impossible at bare/CPE. Figure 11A evidently showed the CV curves obtained at bare/CPE (curve a) and polysorbate/CPE (curve b) for CC and HQ (10.0 μM) in PBS (0.2 M, pH 7.4). At bare/CPE we observed a broad shaped overlain voltammogram and the merged oxidation potential was seen at 0.1736 V, which is of no practical importance. However, at polysorbate/CPE there is a drastic separation of oxidation signals of both the analytes. The oxidation potentials of CC and HQ were situated at 0.153 V and 0.025 V respectively, the difference in the oxidation potential of both the analyte is found to be 0.128 V. This result was more than enough for the determination of these isomers in a binary mixture. On the other hand, these obtained results were again examined by ultra-sensitive differential pulse voltammetry (DPV) method with the merit of absence in the background current. Figure 11B showed the oxidation signals of CC and HQ were indistinguishable at bare/CPE (curve a) and the merged oxidation potential was observed at 0.125 V. However, as expected the polysorbate/CPE (curve b) exhibits the selective separation in the oxidation potentials of CC and HQ (50.0 μM each), the resolved oxidation potentials were located at 0.108 V and − 0.016 V respectively. The difference in oxidation potential of the CC and HQ was 0.124 V. Therefore, it can be concluded a simultaneous detection of hazardous CC and HQ can be achieved at polysorbate/CPE by both CV and DPV techniques. Accordingly, due to the electroactive monolayer formed on the modified electrode facilitates the easier oxidation of HQ than CC, the oxidation potential of HQ shifts to lower potential scale and oxidized well before reaching the oxidation potential of CC which leads a successful separation of these targeted analytes^62^.

### Interference study and analytical application

**Figure 12:**
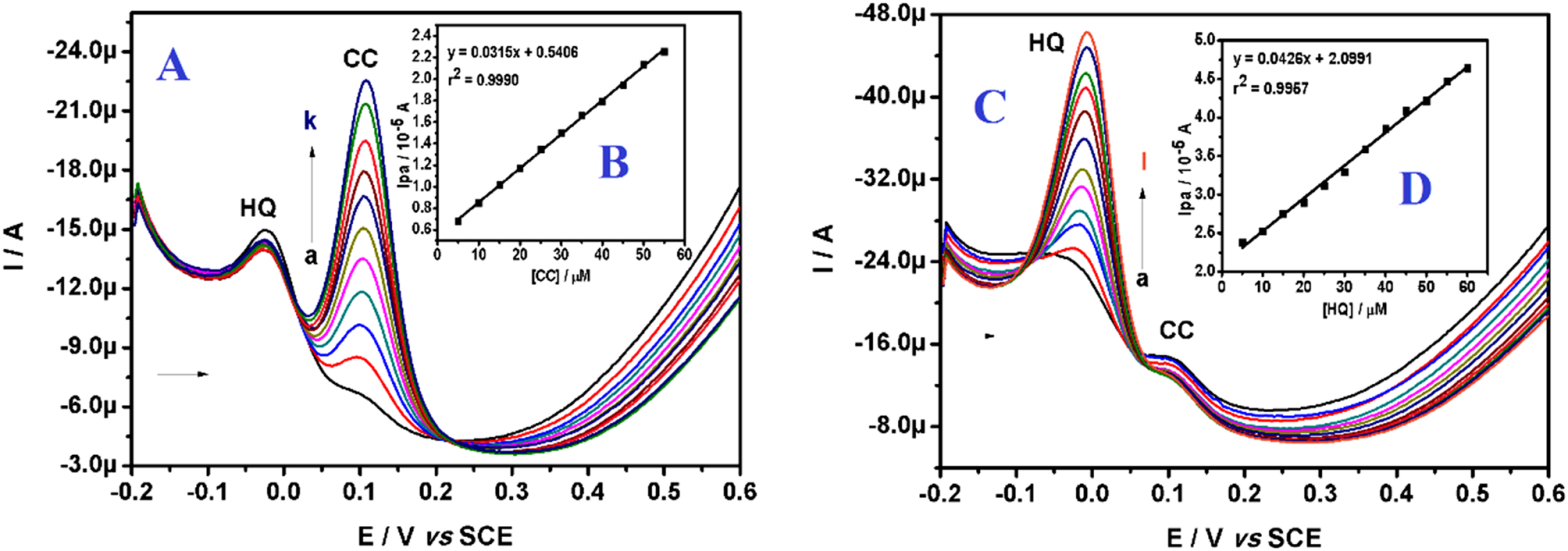
(**A**) DPVs of varying concentration of CC in presence of 20.0 μM HQ at polysorbate/CPE in buffer (0.2 M PBS; pH 7.4). (**B**) Linear plot of Ipa versus concentration of CC. (**C**) DPVs of varying concentration of HQ in presence of 20.0 μM CC at polysorbate/CPE in buffer (0.2 M PBS; pH 7.4). (**D**) Linear plot of Ipa versus concentration of HQ.

In order to evaluate the selectivity of the proposed modified electrode, the interference study was carried out by keeping the concentration of one analyte constant and varying the concentration of other one in a binary mixture^75^. The Fig. 12A shows, as the concentration of CC was varied in a linear range of 5.0 to 55.0 μM by keeping the HQ concentration constant, we can observe an increase in peak current due to an increase in the concentration of CC only. On the other side, the increasing concentration of HQ (5.0 to 60.0 μM) results in the increased peak current of HQ only, when the concentration of CC was kept constant (Fig. 12C). The relationship of peak current and concentration for both the analytes were linear as showed in inset Fig. 12B,D. The corresponding linear regression equation can be written as below:

Ipa (10^-5^A) = 0.0315 (C_0_ μM/L) + 0.5406, (r^2^ = 0.9990) (for CC; HQ constant).

Ipa (10^-5^A) = 0.0426 (C_0_ μM/L) + 2.0991, (r^2^ = 0.9957) (for HQ; CC constant).

To evaluate the analytical applicability of the polysorbate/CPE, the impact of various potentially interfering substances was studied by adding them into the binary mixture of CC and HQ (50.0 μM) in PBS (0.2 M, pH 7.4)^76–78^. It can be noted from Table 5 that the addition of tenfold (0.5 mM) of different substances such as glucose, sucrose, lactose, glycine, ascorbic acid, citric acid, oxalic acid, sodium chloride, potassium chloride, ammonium chloride and calcium sulphate does not affect the determination of CC and HQ at polysorbate/CPE. The change in current signal was not exceeded 5.0%, reflecting the selectivity of the proposed electrode. The determination of CC and HQ in tap water sample was tested and the obtained results were tabulated in Table 6. When a known amount of CC was added to the tap water sample, a recovery of 96.0% to 100.22% was obtained. Similarly, when a known quantity of HQ was added to the tap water sample, a good recovery of 97.0% to 100.88% was observed. Overall, these gathered results can be accepted and it reflects that, the polysorbate/CPE could be successfully applied for the determination of CC and HQ in real samples without any interferences.

**Table 5:** Effect of various interferents on the determination of binary mixture of CC and HQ (50.0 μM) at polysorbate 80/CPE.

Interferents	Current change (%)	
	CC	HQ
Glucose	2.11	2.44
Sucrose	1.98	2.20
Lactose	2.68	2.86
Glycine	2.31	2.82
Ascorbic acid	3.80	3.92
Citric acid	1.93	2.07
Oxalic acid	2.42	2.22
Sodium chloride	1.74	2.10
Potassium chloride	1.82	2.04
Ammonium chloride	1.88	1.98
Calcium sulphate	1.79	1.94

**Table 6:** Results obtained for CC and HQ determination in tap water sample at polysorbate/CPE (n = 3).

Sample	Added (μM)	Found (μM)	Recovery (%)
CC	3.0	2.88	96.0
	6.0	5.96	99.33
	9.0	9.02	100.22
	12.0	11.89	99.08
HQ	3.0	2.91	97.0
	6.0	6.04	100.66
	9.0	9.08	100.88
	12.0	12.08	100.66

## Conclusion

To fabricate an electrochemical sensing interface, it is significant to understand the sensing mechanism and molecular level prediction of the modifier. We investigated the redox reactive sites and locations of energy levels of polysorbate 80 molecule by advanced density functional theory (DFT)-based quantum chemical modelling to investigate the redox reactive sites. From the results, it’s well known that the high values of HOMO orbital energy define the ability of molecule to donate electron. While, the low LUMO energy values explain the acceptor ability. In polysorbate 80 molecule, the HOMO distribution density localized on the C=C group in the carbonic chain while the LUMO density localized particularly in the –O–O– group in the carbonic chain. From the experimental results, a high reactivity performance was observed at modified electrode interface for the determination of dihydroxy benzene isomers. The potential excipients do not have significant impact on the electroanalysis. The fabrication of polysorbate 80 modified electrode with the novel prediction of redox reactive sites may lay a new platform for molecular level understanding of the modifiers.
